# Artificial Intelligence in Orofacial Pain: Diagnostic and Predictive Performance Across Machine Learning and Deep Learning Models

**DOI:** 10.3390/diagnostics16121801

**Published:** 2026-06-11

**Authors:** Laura Iosif, Marina Imre, Andreea Gabriela Wagner, Ana Maria Cristina Țâncu, Andreea Cristiana Didilescu, Hendrik Simon Brand, Andra-Ana-Maria Cîmpean, Radu Ilinca, Lucian Toma Ciocan, Vlad Gabriel Vasilescu

**Affiliations:** 1 Department of Prosthodontics, Faculty of Dentistry, Carol Davila University of Medicine and Pharmacy, 010232 Bucharest, Romania; laura.iosif@umfcd.ro (L.I.); marina.imre@umfcd.ro (M.I.); anamaria.tancu@umfcd.ro (A.M.C.Ț.); 2 Department of Embryology, Faculty of Dental Medicine, Carol Davila University of Medicine and Pharmacy, 8 Eroii Sanitari Boulevard, 050474 Bucharest, Romania; andreea.didilescu@umfcd.ro; 3 Department of Oral Biochemistry, Academic Centre for Dentistry Amsterdam (ACTA), VU University of Amsterdam and University of Amsterdam, Gustav Mahlerlaan 3004, 1081 LA Amsterdam, The Netherlands; h.brand@acta.nl; 4 Faculty of Dentistry, Carol Davila University of Medicine and Pharmacy, 17–23 Calea Plevnei, District 5, 010221 Bucharest, Romania; andra-ana-maria.cimpean2022@stud.umfcd.ro; 5 Department of Medical Informatics and Biostatistics, Faculty of Dentistry, Carol Davila University of Medicine and Pharmacy, 4–6 Eforie Street, 050037 Bucharest, Romania; 6 Discipline of Dental Prosthesis Technology, Faculty of Dentistry, Carol Davila University of Medicine and Pharmacy, Dionisie Lupu Street, No. 37, District 2, 020021 Bucharest, Romania; lucian.ciocan@umfcd.ro (L.T.C.); vlad.vasilescu@umfcd.ro (V.G.V.)

**Keywords:** oral, maxillofacial pain, artificial intelligence, diagnosis, pain measurement, odontogenic pain, temporomandibular joint disorders, neuropathic pain, neurovascular pain, dental diagnosis

## Abstract

Orofacial pain (OFP) includes a broad spectrum of odontogenic and non-odontogenic conditions with overlapping clinical features that often limit diagnostic accuracy, driving increasing interest in artificial intelligence (AI) as a tool to enhance diagnostic precision and support clinical decision-making. A narrative review was conducted using PubMed/MEDLINE, Scopus, and Web of Science to identify studies (2016–2026) applying AI to the diagnosis, classification, or prediction of OFP in adults. Eligible studies reported at least two diagnostic performance metrics and were thematically grouped into odontogenic and non-odontogenic categories, the latter including musculoskeletal, neurovascular, and neuropathic pain. Twenty studies were included. Neurovascular pain, particularly migraine, showed the most consistent and highest diagnostic performance, likely due to the greater availability of structured clinical data and standardized diagnostic criteria. Musculoskeletal pain, especially temporomandibular disorders, also demonstrated high and reproducible performance. In contrast, odontogenic pain showed lower and more heterogeneous performance, with better results mainly in imaging-based models, while signal- and behavior-based approaches were less robust. Neuropathic pain exhibited moderate to high performance in selected radiomics studies, but overall results remained inconsistent due to phenotypic variability and limited objective biomarkers. Currently, AI shows promising potential in OFP diagnosis, especially for neurovascular and musculoskeletal pain, but clinical translation is limited by data heterogeneity and lack of validation. Progress in clinical practice depends on multimodal datasets and multicenter studies to ensure robust, generalizable tools.

## 1. Introduction

Orofacial pain (OFP), one of the most common pain conditions worldwide with a reported prevalence of 17–26% [1], encompasses a broad spectrum of cranial, oral, and facial pain disorders, while being determined by social, cultural, and demographic predictors, as well as by patients’ dental awareness and access to care [2]. This major public health problem [3] exerts a significant and profound impact on quality of life [4] and continues to represent one of the primary drivers of dental healthcare service utilization [5].

Dental pain is the most frequently reported symptom (57.6%) [6], followed by temporomandibular joint (TMJ) pain [7] (14.8%), facial pain [8] (13.2%), and predominantly inflammatory pain conditions [9]. In parallel, the head and neck represent some of the most commonly affected regions in chronic pain disorders [10], emphasizing the importance of accurate assessment and diagnosis.

However, the diagnosis of OFP remains challenging due to the involvement of multiple anatomical structures, heterogeneous etiologies [11], variable patterns of pain referral and presentation (acute or chronic), and the lack of consensus regarding differential diagnostic criteria [12]. In addition, local mechanical factors, psychological influences, and comorbidities further contribute to its clinical complexity [13]. Given this multifactorial nature, standardized classification is essential for improving diagnostic accuracy and clinical decision-making. Accordingly, the International Classification of Orofacial Pain (ICOP), which was established in 2020, is the first system specifically dedicated to this domain, is structured hierarchically, and encompasses dentoalveolar and related tissue pain, muscular pain, TMJ disorders (TMJDs), neuropathic pain involving cranial nerves, headache-like pain, and idiopathic OFP [14]. A subsequent clinically derived sub-classification, based on the ICOP framework, further classified OFP into odontogenic and non-odontogenic categories, with the latter encompassing musculoskeletal, neurovascular, and neuropathic pain [15,16,17], which has been adopted as the basis for the current research.

Building on this classification, our review was motivated by the persistent challenge of quantifying pain, which remains difficult even with the currently available techniques, as pain is inherently subjective and cannot be reliably measured using a single method. Its assessment mostly relies on patient-reported pain sensations, typically captured through scales such as the Visual Analog Scale (VAS), Verbal Rating Scale (VRS), and Numerical Rating Scale (NRS) [18], while being complemented by clinical examination, imaging, and laboratory investigations that provide indirect information relevant to diagnosis. However, all of these approaches remain limited by their dependence on subjective perception and the indirect relationship between the actual pain experience and measured parameters [19,20,21]. Consequently, the development of objective and reproducible approaches, including quantitative neurophysiological and signal-based methods [22,23,24], has become essential for improving diagnostic accuracy, differential diagnosis, and prognostic evaluation.

In OFP, these approaches include techniques such as electroencephalography (EEG) [25], functional magnetic resonance imaging (fMRI) [26], trigeminal evoked potentials [27], electromyography (EMG) [28], quantitative sensory testing (QST) [29], autonomic measures including heart rate variability (HRV) [30], skin conductance (galvanic skin response) [31], pupillometry [32], and facial expression recognition using computer vision-based analysis [33], which do not directly measure pain but rather assess neural activity, reflex responses, and autonomic or computational signals associated with nociceptive processing.

There are robust signs that artificial intelligence (AI), encompassing machine learning (ML) and deep learning (DL) algorithms, may play an increasing role in pain assessment in the coming years, particularly through the integration and interpretation of datasets. Starting from AI-based decision-support systems developed to assist in the diagnosis of OFP associated with TMJDs in clinical decision-making [34], more recent approaches rely on behavioral and neurophysiological frameworks for automated pain assessment. Accordingly, behavioral AI approaches include, among others, facial expression recognition and natural language processing (NLP), both of which have been operationalized through automated image classification, feature extraction, and linguistic pattern analysis [35,36]. In parallel, AI-based neurophysiological pain detection relies on the analysis of biological signals such as electroencephalography (EEG), electromyography (EMG), electrodermal activity (EDA), among other physiological recordings [37]. More recent developments emphasize multimodal AI strategies that integrate behavioral and neurophysiological data to improve classification accuracy and robustness in pain assessment.

Nevertheless, despite the increasing integration of AI methodologies in OFP research, their clinical applicability and technological maturity remain insufficiently established in evidence-based practice, particularly regarding their diagnostic consistency across heterogeneous pain conditions of the orofacial region. To the best of our knowledge, the diagnostic and predictive performance of AI algorithms—including both ML and DL models—across the main categories of OFP, tested on real patients or on clinical data derived from electronic health records (EHRs), has not yet been comprehensively addressed in the literature, representing a critical gap in our current understanding of which AI approaches hold genuine potential for clinical translation in one of the most diagnostically challenging and, consequently, therapeutically underserved domains of dental medicine. Therefore, the aim of this study was to review and critically evaluate AI-based approaches applied to the diagnosis, prediction, and assessment of OFP in adult human subjects, focusing exclusively on studies that meet predefined criteria regarding diagnostic performance metrics, in order to assess their clinical effectiveness, limitations, and comparative performance across different pain subdomains.

The hypothesis was that diagnostic performance varies between odontogenic and non-odontogenic OFP conditions, with the latter including musculoskeletal, neurovascular, and neuropathic pain categories. Accordingly, the main objective was to identify domains in which AI demonstrates the highest diagnostic performance and to highlight areas where current evidence remains limited, in order to better define existing research gaps and guide future clinical research.

## 2. Materials and Methods

### 2.1. Study Design

This work was designed as a narrative review aimed to synthesize the current evidence on the following topic: OFP conditions, further classified into odontogenic, musculoskeletal, neurovascular, and neuropathic subtypes, each of them being evaluated by AI models. The review focused on original articles resulted from clinical and observational research, addressing diagnostic, prediction, and clinical implications.

### 2.2. Search Strategy

A comprehensive literature search was conducted in three major international electronic databases: PubMed/MEDLINE, Scopus, and Web of Science. The search covered a ten-year period (2016–2026) to ensure inclusion of the most recent evidence in the field. A uniform, keyword-based search strategy was applied across all databases to maintain consistency, irrespective of indexing systems.

The search strategy combined terms related to AI (including “artificial intelligence”, “machine learning”, “deep learning”, and “convolutional neural networks (CNN)”, as well as the abbreviations AI, ML, DL, and CNN) with terms related to orofacial and stomatognathic pain conditions. These included all major etiological categories of pain, namely odontogenic pain, periodontal pain, and musculoskeletal pain including TMJD, neuropathic pain, and neurovascular pain. To ensure comprehensiveness and reduce the risk of missing relevant studies, additional articles were identified through manual screening of the reference lists of all included studies and relevant systematic reviews.

The following items were introduced in the query box ul of each database, as follows: (“artificial intelligence” OR “machine learning” OR “deep learning” OR “neural networks” OR “neural network” OR “radiomics” OR “computer vision” OR “predictive model” OR “predictive modeling” OR “diagnostic model” OR “classification model”) AND (“orofacial pain” OR “facial pain” OR “dental pain” OR “toothache” OR “pulpitis” OR “acute pulpitis” OR “dental pulp disease” OR “periapical abscess” OR “periapical periodontitis” OR “periodontitis” OR “periodontal disease” OR “acute periodontal pain” OR “pericoronitis” OR “acute pericoronitis” OR “pericoronitis” OR “odontogenic pain” OR “odontogenic infection” OR “cracked tooth syndrome” OR “dentine hypersensitivity” OR “TMJD” OR “temporomandibular disorder” OR “temporomandibular joint disorder” OR “myofascial pain” OR “jaw muscle pain” OR “masticatory muscle pain” OR “bruxism” OR “trigeminal neuralgia” OR “postherpetic neuralgia” OR “post-traumatic trigeminal neuropathic pain” OR “persistent idiopathic facial pain” OR “PIFP” OR “burning mouth syndrome” OR “orofacial migraine” OR “migraine” OR “cluster headache” OR “tension headache” OR “sinusitis” OR “maxillary sinusitis” OR “oral cancer pain” OR “oral mucositis” OR “salivary gland pain” OR “salivary gland disease”).

The detailed search strategy is summarized in Table 1.

### 2.3. Eligibility Criteria

Inclusion criteria were:

- Studies involving humans with OFP, regardless of etiology.
- Studies employing at least one AI method (including ML, DL, or CNN) for the diagnosis, classification, assessment, or prognosis of OFP.
- Studies reporting at least two diagnostic performance metrics, such as sensitivity, specificity, area under the curve (AUC), precision, recall, or F1 score, or studies reporting overall diagnostic accuracy as a standalone performance indicator
- Original full-text studies published in English, including retrospective and prospective designs, diagnostic accuracy studies, cohort studies, and experimental studies.

The following exclusion criteria were applied:

- Studies involving pediatric or neonatal patients.
- Studies focusing on AI applications in non-stomatognathic pain conditions (e.g., low back pain, visceral pain, or general postoperative pain without an orofacial component).
- Studies that did not employ an actual AI model (e.g., purely theoretical discussions, editorials, letters to the editor, conference abstracts, or study protocols).
- Studies lacking quantifiable AI performance metrics, which were considered only for narrative discussion if applicable.
- Reviews, meta-analyses, and narrative review articles.

### 2.4. Study Selection

The identified records were imported into a bibliographic management software, Zotero Version 9.0.4. (Corporation for Digital Scholarship, Fairfax, VA, USA), where duplicates were automatically removed. Two independent reviewers (A.G.W. and A.-A.-M.C.) screened titles, abstracts, and reference lists to identify potentially eligible studies. Any disagreements between reviewers were resolved through discussion or, when necessary, by consultation with a third reviewer (L.I.). Full-text articles were retrieved and assessed for eligibility according to the predefined inclusion criteria.

A total of 20 studies were identified, screened, and ultimately included in the present review. Data extraction included information on methods for quantifying dental pain and disease, characteristics of training and testing datasets used in ML or DL models, diagnostic performance outcomes, and comparisons with clinician-based diagnoses.

From each included article, data on study design, population, pain type(s) investigated, diagnostic criteria, and key findings relevant to the proposed pain classification were extracted using a standardized form. Findings were summarized qualitatively and organized narratively according to four predefined categories: odontogenic and non-odontogenic pain, with the latter including musculoskeletal, neurovascular, and neuropathic pain.

To assess the methodological transparency of the present review, the ROBIS (Risk Of Bias In Systematic Reviews) tool was applied [38] acknowledging that, although originally developed for systematic reviews, its domains are relevant for evaluating key methodological aspects of any evidence synthesis, including narrative reviews. The results of this assessment are presented in Supplementary Table S1.

## 3. Results and Discussion

In contemporary clinical practice, OFP continues to place a considerable strain on both diagnostic processes and therapeutic management. Its heterogeneous origins—including odontogenic (dental and periodontal), maxillary, musculoskeletal, salivary-gland-related, tumor-related, neuropathic, and neurovascular sources—frequently converge into overlapping symptom patterns that obscure the underlying pathology. This diagnostic puzzle-like ambiguity and the inherent difficulty of differential diagnosis directly influence treatment selection and long-term prognosis, reinforcing once more the need for high-quality evidence capable of guiding clinical decision-making and, importantly, for innovative technologies capable of enabling highly accurate diagnostic assessment by detecting even the most subtle variations in nociceptive expression.

**Figure 1:**
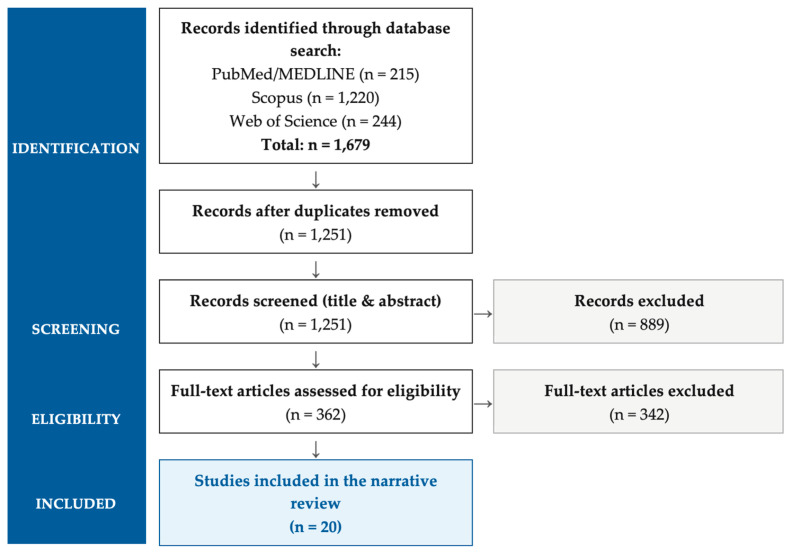
PRISMA flowchart of the study selection process.

**Figure 2:**
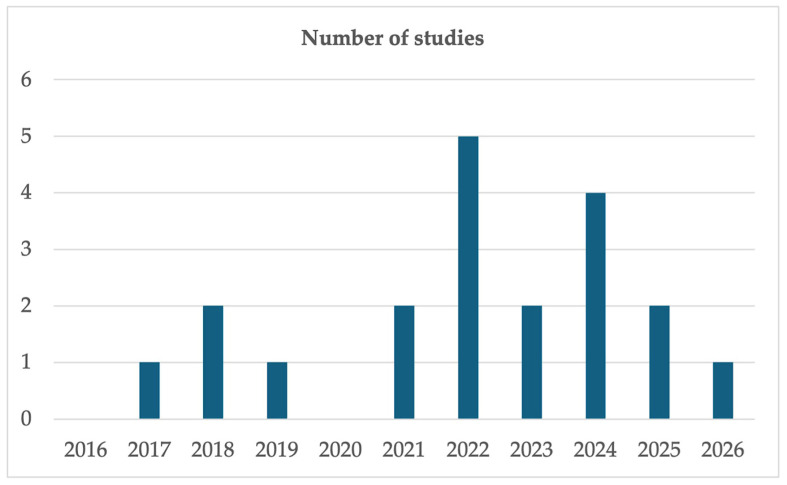
Distribution of included studies by year on AI-based diagnostic and management approaches for OFP.

Regarding this, the initial search identified 1679 records across the three medical databases. Further, only 1251 remained after removing 428 duplicates, and following title and abstract screening, 889 records were excluded, leaving 362 full-text articles for eligibility assessment. Ultimately, 20 studies met the methodological rigor required for inclusion; the complete selection process is presented in the PRISMA flowchart in Figure 1, while their numerical distribution across the investigated decade is shown in Figure 2.

**Figure 3:**
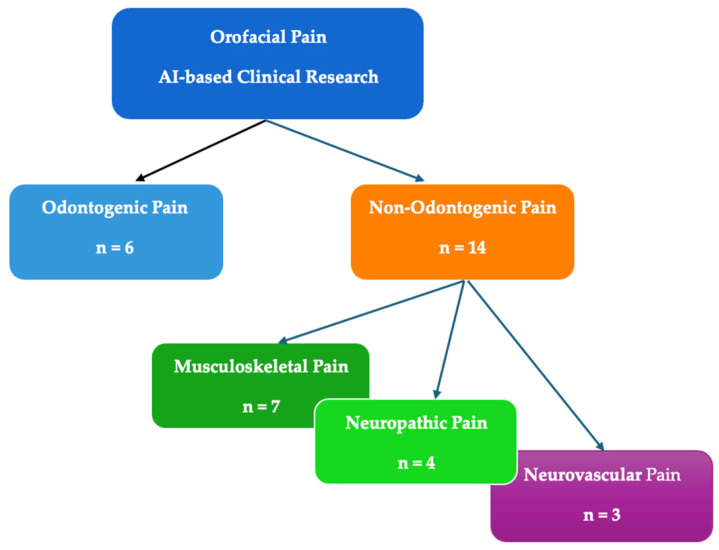
Hierarchical distribution of AI-based clinical research in OFP.

Among these, odontogenic pain accounts for six studies, while within the non-odontogenic category, musculoskeletal pain represents the most frequently investigated condition (seven studies), followed by neurovascular (four studies) and neuropathic pain (three studies), as hierarchically illustrated in Figure 3. Notably, no studies were identified that specifically address the use of AI for diagnostic or predictive analysis of tumor- or salivary gland-related sources of OFP.

### 3.1. Odontogenic Pain

Dental, or odontogenic pain, represents one of the most highly prevalent and clinically significant painful conditions within the spectrum of OFP disorders [39], exerting a substantial impact on patients’ quality of life [40] and functional capacity.

From a neurobiological origin, odontogenic pain arises through activation of sensory neurons of the trigeminal nerve, which transmit nociceptive signals from injured or inflamed dental tissues, most commonly involving the dentine–pulp complex, frequently as a consequence of dental caries, followed by periodontal structures [41].

From an etiological standpoint, odontogenic pain may be broadly categorized into pulpal, periapical, periodontal, and structural origins, each representing a distinct primary site of tissue involvement and an associated pathological process within the dental apparatus [42]. Pain of pulpal origin is commonly linked to inflammatory or degenerative changes within the dental pulp, where initial irritation triggers an inflammatory cascade that, if left unresolved, may progress toward irreversible pulp damage, eventual necrosis, and subsequent extension of the pathological process into the periapical region and surrounding alveolar bone. However, dental pain associated with pulpal involvement remains diagnostically challenging due to the limited accessibility of the dental pulp to conventional clinical testing methods, the often nonspecific and poorly localized nature of its symptomatology, and the potential presence of referred pain patterns arising from adjacent periodontal structures, which may further obscure the accurate identification of the primary source of discomfort [43,44,45].

A clinician’s ability to rapidly and accurately diagnose such conditions is essential for effective patient management of pain and of its source; however, several studies have reported that diagnostic errors in these cases remain relatively frequent [46]. Recent advances in the diagnosis and management of odontogenic pain have been driven by improvements in clinical assessment methods, imaging techniques, and therapeutic strategies, leading to more accurate and targeted patient care. In terms of diagnosis, enhanced pulp vitality testing, including electric pulp testing, optical pulp scanning [47], laser Doppler flowmetry (LDF) [48], pulse oximetry (PO) [49], oxygen saturation [50], thermal tests, infrared thermography [51] and also infrared spectroscopy [52], has been complemented by more reliable sensibility and vitality assessment methods, while cone–beam computed tomography (CBCT) has significantly improved the detection of periapical pathology, root fractures [53], and complex endodontic conditions that may not be visible on conventional radiographs. Additionally, a better understanding of pain mechanisms, including inflammatory pathways and peripheral sensitization, has refined the differential diagnosis between pulpal and periapical conditions.

From a therapeutic perspective, contemporary endodontic pain management is increasingly oriented toward minimally invasive and preventive strategies, including vital pulp therapy procedures such as pulp-capping and pulpotomy [54], which aim to preserve pulp vitality by intervening at early stages of disease progression. In parallel with this shift toward early intervention, preservation, and improved preoperative pain management, AI has gained increasing prominence in dentistry, particularly in the context of detecting early pathological signals and supporting clinical decision-making through data-driven approaches [55]. Within this field, ML especially has demonstrated strong potential for pattern recognition and predictive analytics, with performance that continues to improve as new datasets are integrated [56]. Although pain remains inherently subjective and cannot be directly quantified, ML-based models have recently been applied to the prediction of postoperative pain in cases of irreversible pulpitis, with the aim of anticipating patient outcomes and optimizing postoperative pain management strategies.

The importance of such advances is underscored by the fact that severe aching pain may persist following endodontic interventions, such as root canal therapy or apicectomy, and in a subset of patients this may evolve into a chronic neuropathic pain state [57]. Our research identified multiple supervised ML algorithms trained in 2026 by de Jesus Freitas et al. [58] to predict the occurrence of postoperative pain in 345 patients, such as Logistic Regression (LR), Support Vector Machine (SVM), Gradient Boosting (GB), Random Forest (RF), Decision Tree (DT), K-Nearest Neighbors (KNNs), Adaptive Boosting (AdaBoost), and Multilayer Perceptron (MLP). Among the eight predictive models, all of which demonstrated good discrimination of pain outcomes, particularly within the first 24 h, the LR model was able to correctly identify approximately eight out of every ten patients who subsequently reported pain, a level of performance that is especially clinically relevant as it enables clinicians to anticipate which individuals are at higher risk of developing immediate discomfort and to make timely adjustments in pain management strategies or consider supplementary interventions. For pain persisting beyond 72 h, the SVM model exhibited even stronger predictive performance, effectively identifying the majority of cases in which pain extended beyond the first postoperative day, thereby supporting the implementation of individualized preventive strategies in special cases, while taking into account that patient age and sex were consistently shown to be the most influential predictors across models, contributing to improved symptom control, a reduction in complications, and an overall enhancement of the patient’s treatment experience. Another aspect that also drew attention was that the RF model achieved rather moderate predictive performance for both evaluated time intervals, a finding that is consistent with the previous results reported in 2023 by Nosrat et al. [59]. The same ML algorithm has also been investigated in the context of flare-up prediction, with flare-ups being defined as moderate-to-severe pain with or without swelling occurring within 14 days of the initiation of nonsurgical retreatment and resulting in an unscheduled treatment appointment. In that study, the RF model yielded values of 0.82 for accuracy, 0.49 for sensitivity, 0.13 for precision (positive predictive value), and 0.83 for specificity, while receiver operating characteristic (ROC) and precision–recall (PR) curves were generated and the corresponding area under the curve values were calculated; overall, however, the model demonstrated weak predictive performance. In this regard, the consistently low precision values and weak flare-up prediction performance reported across both studies raise concerns regarding the clinical reliability of RF-based models in this context, underscoring the need for external validation on larger and more diverse patient cohorts.

In 2020, another study included in this review also used a RF-based model; this study was based on the observation that any response to pain is governed by a complex interplay of metabolic alterations, shifts in hormonal equilibrium, and dynamic changes in autonomic nervous system activity. In this context, Teichmann et al. [60] aimed to investigate the feasibility of detecting brief pain sensations through the analysis of cardiorespiratory signals recorded during dental procedures, with twenty patients being exposed to pain-inducing dental stimuli and simultaneously reporting their pain episodes by pressing a push-button device, while their cardiorespiratory parameters were continuously monitored in parallel. The results showed that, at the optimal operating point defined by the maximum harmonic mean between sensitivity and specificity, the algorithm achieved approximately 87% sensitivity and 63% specificity, indicating that the detection of brief pain events based on physiological signals is feasible; however, the authors explicitly emphasize that the overall performance remains relatively low and is likely insufficient for clinical use. They further note that the operating threshold along the ROC curve could be adjusted to favor either sensitivity or specificity, although this would involve a trade-off rather than a true improvement in overall performance.

Alongside intelligently monitored cardiorespiratory parameters, as in Teichmann’s study [60], the feasibility of detecting dental pain through automated, intelligent monitoring of facial expressions has been extensively investigated over the past decade. This technology, widely employed in other non-medical fields, may potentially be adapted particularly for patients experiencing communication difficulties due to cognitive decline or other conditions, who often find it challenging to verbally express their needs, discomfort, or pain. An exploratory investigation evaluated the use of Automated Face Coding (AFC) software to identify pain-related facial expressions and emotional states in the context of dental pain screening [61]. The study tested the null hypothesis that the AFC system would not differentiate between facial expressions recorded during pain and those recorded after pain relief. Although self-reported pain levels decreased significantly following intervention, the AFC system did not detect corresponding changes in facial expressions or emotional states. These findings highlight the limited performance of the current AI-based AFC system in reliably capturing pain-specific facial cues in this clinical context. To date, both physiological signal- and facial expression-based approaches failed to reach clinically meaningful performance thresholds, limiting their translational potential.

Objective dental pain assessment using neuroimaging techniques, focused on brain-based detection of dental pain, has been the subject of numerous scientific debates; however, only a limited number of studies have incorporated AI methods in a clinically applicable manner. One of the few clinical studies employing DL approaches for real-time pain detection is that of Hu et al. [62], who developed the CLARAi framework integrating functional near-infrared spectroscopy (fNIRS), AI, and augmented reality (AR) for the real-time detection and localization of clinical pain directly from brain activity. Within this framework, multiple neural network architectures were tested, including artificial neural networks (ANNs), CNN, recurrent neural networks (RNNs), and long short-term memory (LSTM) models, in order to classify hemodynamic responses into pain and no-pain states, as well as to perform left- versus right-sided pain localization. Among these, ANN-based models achieved the highest overall performance for pain detection, reaching an accuracy of 80.37% and a positive likelihood ratio of 2.35, whereas CNN-based architectures, particularly a six-layer CNN, performed best in the three-class localization task with an accuracy of 74.23%. In this regard, despite these promising results, the small sample size and the moderate sensitivity reported across architectures suggest that the CLARAi framework remains insufficiently robust for reliable clinical translation.

DL models were also applied for objective dental pain assessment using infrared imaging technology, as reported by Haddad et al. [63] in 2022. The authors address the limitations of conventional diagnostic imaging in inflammatory dental pain by introducing infrared thermography as an imagistic method capable of capturing functional rather than structural alterations, specifically reflecting underlying microcirculatory dynamics, including local vasomotor responses associated with inflammatory processes [64]. Thermographic data were acquired from 76 adult volunteers, including individuals with inflammatory toothache and subjects who were clinically healthy, all of whom also underwent radiographic and clinical examination. The thermographic images were processed using four CNN architectures—MobileNetV2, InceptionV3, ResNet50V2, and ResNet101V2—selected for automated image-based classification through hierarchical feature extraction. Performance varied across models, with MobileNetV2 achieving superior classification results in both frontal and lateral image sets, while ROC curve analysis indicated better discrimination for frontal thermographic acquisitions.

In the field of periodontal pain diagnosis, scientific reports regarding the use of AI tools are extremely scarce, a fact not least attributable to the relatively rare occurrence of spontaneous pain in periodontal conditions. A single study by Teichmann et al. [65], published in 2018, was identified in our search, with the authors employing the same methodological approach later used by others [61], namely the detection of brief pain sensations through the analysis of cardiorespiratory signals recorded, in this case, during experimentally induced pain via periodontal probing in patients with periodontitis. The study also used ML, specifically a RF classifier, to detect periodontal pain sensations from physiological signals. Forty-seven subjects underwent periodontal probing and reported pain episodes via a push-button, while autonomic indices were extracted from simultaneously recorded ECG and PPG (photo plethysmogram) signals. The RF model was used for the detection of periodontal pain sensations from these physiological signals, achieving optimal performance during validation and yielding a sensitivity of 71% and a specificity of 70% on an independent test set, results which can be considered reasonably acceptable in the context of physiological signal-based periodontal pain detection.

### 3.2. Non-Odontogenic Pain

- Musculoskeletal pain

Among the most common pain-generating conditions within the musculoskeletal category of the orofacial region, TMJDs are the most prevalent. These represent one of the leading causes of OFP musculoskeletal pain encompassing a group of disorders characterized by pain and/or impaired function of the masticatory muscles, the TMJs, and associated structures [66,67], and a range of subjective clinical features [68,69].

Practitioners generally find the causes and symptoms of TMJDs to be quite nonspecific and amenable to multiple diagnoses, often with overlapping symptoms of other musculoskeletal and neurological conditions [70]. The causes of TMJDs are multifactorial, involving a combination of physical and psychosocial factors [71,72]. Occlusal discrepancies, parafunctional habits, psychosocial stress, hormonal influences, and genetic predispositions have all been identified as contributing factors [73].

Acknowledging that TMJD encompasses both structural and biopsychosocial components, the DC/TMD (Diagnostic Criteria for Temporomandibular Disorders) employs a dual-axis assessment approach for diagnosis, that integrates standardized clinical examination for joint- and muscle-related disorders with validated instruments assessing behavioral, psychological, and psychosocial status [74,75,76]. In addition to clinical examination, multiple supplementary diagnostic approaches are utilized to provide a comprehensive evaluation of TMJD. Imaging techniques play a pivotal role in the diagnostic process, offering a more nuanced visualization of the TMJ structure and integrity, in order to relate symptomatic TMJ and OFP. On the other hand, the nonspecific physical symptom scales for TMJ-related pain (e.g., TMJD Pain Screener, 3Q/TMJD Screener) [77,78] are used to assess patients’ nonspecific physical symptoms and depression levels, respectively, with the depression assessment including seven additional items. Cut-off values on these scales categorize the severity of depression and nonspecific physical symptoms as normal, moderate, or severe.

In recent years, AI has emerged as a promising solution to these diagnostic challenges. ML and DL algorithms can process complex multimodal data including imaging, clinical, and psychosocial data to enhance diagnostic accuracy, consistency, and efficiency [79,80]. AI-assisted imaging has shown strong performance in detecting disk displacement, degenerative changes, and other TMJDs, while predictive models based on symptoms, wearable sensors, and AI-driven decision-support tools are broadening diagnostic capabilities [81,82]. DL architectures, especially CNNs, facilitate automated segmentation, feature extraction, and classification of both bone and soft-tissue structures, providing an objective and reproducible supplement to human interpretation [83]. However, this category of diagnostic tools relate TMJD to modification in radiologic density and shape of anatomical features and not to pain.

Although limited in absolute number, this category represented the largest proportion of eligible studies identified in the reviewed literature (Figure 3). In contrast, contemporary approaches to musculoskeletal pain diagnosis increasingly rely on symptom- and data-driven predictive models, as well as AI-assisted screening and decision-support tools, which are currently considered among the most widely adopted and reliable methods.

With the growing use and accessibility of electronic health record (EHR) data, ML methods have been widely applied to generate data-driven clinical predictions [84]. Numerous studies have applied ML for clinical predictions, such as assessing symptom severity in mental health care [85], diagnosing common headaches [86], and forecasting fertility [87]. Several studies developed models to diagnose OFP from patient pre-interview questionnaires. Specifically, in 2013, McCartney et al. [88] first developed and applied a neural network (NN)-based system to diagnose facial pain syndromes using patients’ self-reported assessments. Limonadi et al. [89] subsequently implemented this NN model in a separate cohort of 143 patients, utilizing data from an 18-item binary (yes/no) questionnaire to support diagnosis. They reported favorable performance for several of the seven diagnostic categories evaluated.

Within the selected timeframe of our study, a key contribution to the field of musculoskeletal facial pain diagnosis was made in 2018 by Nam et al. [90], who compared 29 patients with TMJD-mimicking conditions and patients with genuine TMJD based on chief complaints, clinical history, and objective examination findings, integrating symptom-based diagnostic data with NLP and achieving a diagnostic accuracy of 96.6% in differentiating between the two entities, while also identifying distinct differences in symptom-related language use between groups. Although TMJD-mimicking conditions are rare and may arise from infections, neoplasms, developmental disorders, or genetic abnormalities, their accurate recognition remains clinically essential due to its impact on prognosis and treatment outcomes. In this regard, the high diagnostic accuracy reported should be interpreted with caution, given the small sample size and the rare clinical prevalence of TMJD-mimicking conditions.

In 2021, comparable performance was reported by Nocera et al. [91], who achieved accuracy rates ranging from 75.60% to 96.89% in detecting the five most frequent OFP disorders based on age, pain severity, and maximum mouth opening, using multiple ML approaches and comparing them with a simplified high frequency value (HFV) algorithm designed to emulate expert diagnostic reasoning, while also noting that reliance on patient questionnaires as the basis of expert judgment may introduce bias and emphasizing the need for future research to develop more robust predictive questionnaires for OFP diagnoses, with a focus on improving question formulation, clarity, and diagnostic relevance.

Furthermore, in 2022, Kreiner et al. [92] tested the performance of a novel NN, Multilayer Perceptron, with diagnostic capabilities in OFP and TMJDs. As for the results, they found that the diagnostic accuracy of the AI was superior to that of the general dental clinicians. Overall, their study demonstrates strong evidence that an artificial NN can perfectly assist dental clinicians in accurately diagnosing OFP and dysfunction, including TMJD, neuropathic, neurovascular, and referred cardiac pain. In this context, while both studies [91,92] report strong diagnostic performance, the reliance on questionnaire-based data and the absence of external validation cohorts limit the generalizability of these findings to broader clinical populations.

Other models, as presented by Yildiz et al. [93], based on bagging and Multivariate Adaptive Regression Splines (MARS), achieved up to 89.7% accuracy and an AUC of 0.939 in predicting TMJD presence from clinical variables (pain intensity, maximum mouth opening (MMO), TMJ lateral excursion movements, pressure pain threshold values of the masseter and temporalis anterior muscles). These findings emphasize the important role of AI in helping inexperienced clinicians make a preliminary detection of TMJDs.

More recently, various screening tools and clinical examinations for detecting TMJDs have been developed to address gaps in the diagnosis, mainly supporting non-specialized dental practitioners who have demonstrated less skills to perform diagnostic and therapeutic procedures related to TMJD. Zaat et al. [94], in 2024, combined symptom-based diagnostic data with NLP, enhancing diagnostic accuracy among non-specialist dentists. Using a Decision Tree-based ML, they presented 84% accuracy in the diagnosis of joint pain, and 78% accuracy for classifying myofascial pain among general practitioners. However, moderate sample sizes and restriction to specific clinical variables suggest that these models require further refinement before routine clinical use.

Another important development in this field was demonstrated by Araujo et al. [95], who reported that the PAINe chatbot, an AI-based virtual assistant, was able to differentiate TMJDs in patients presenting with tooth pain with an accuracy of 86%, while also providing clear responses and effective user interaction.

Building on this line of research, AI-driven screening tools and mobile applications are increasingly being developed for teledentistry and chairside use, integrating questionnaire data, imaging inputs, and predictive models to support preliminary assessment and clinical triage. This trend is exemplified by the myTMJ application described by Lee et al. [96], which combines multimodal data for self-guided screening of jaw pain, headaches, and TMJD, achieving a diagnostic accuracy of 95.5%, exceeding that reported by orofacial pain specialists (90%). Despite these progresses, it should be noted that chatbot- and app-based diagnostic tools introduce additional concerns regarding data privacy, user compliance, and diagnostic accountability that remain largely unaddressed in the current literature.

- Neuropathic pain

Neuropathic pain in the orofacial region represents a chronic and often profoundly debilitating maladaptive expression arising from damage or disease affecting the somatosensory nervous system—most notably the trigeminal nerve. Unlike typical dental pain, which is generally nociceptive in origin, neuropathic pain is distinguished by burning, shooting, or electric shock-like sensations that may occur spontaneously or be triggered by minimal stimuli such as light touch (allodynia) [97,98].

The trigeminal nerve plays a central role in orofacial sensory pathways, and trigeminal neuralgia (TN) represents one of the most prevalent neuropathic pain conditions affecting the head and neck region [99]. This debilitating and lancinating chronic neuropathic condition is characterized by paroxysmal episodes of electric shock-like one sided-facial pain [100]. Modern technologies in the diagnosis and treatment of TN are increasingly shaped by the paradigm of precision medicine, integrating advanced neuroimaging and minimally invasive therapeutic strategies to enhance diagnostic accuracy and optimize patient outcomes, as high-resolution MRI techniques—particularly 3D CISS (Constructive Interference in Steady State) and FIESTA (Fast Imaging Employing Steady-state Acquisition) [101] sequences—enable detailed visualization of neurovascular conflict and significantly improve the detection of subtle vascular compression at the root entry zone of the trigeminal nerve.

In present, emerging DL-based approaches, including CNN and radiomics-driven models, facilitate automated nerve segmentation and classification, reducing observer variability and enabling more objective identification of pathological features. The clinical research of Mulford et al. [102] aimed to determine whether quantitative imaging biomarkers extracted from trigeminal nerve MRI could reliably distinguish between pain-afflicted and pain-free nerves in human subjects. The model demonstrated consistent discriminatory performance, achieving a mean validation accuracy of 78% and an AUC of 0.83, with sensitivity and specificity of 0.82 and 0.76, respectively. Importantly, only 16 radiomics features were retained as highly informative predictors, suggesting the presence of stable imaging-derived signatures associated with neuropathic pain in TN. Nevertheless, the limited sample size and single-center design raise questions about the reproducibility of these imaging-derived signatures across heterogeneous TN populations.

The growing need to simplify and objectify the diagnosis of TN motivated the team of Latypov et al. [103] further to explore the integration of AI algorithms into structural neuroimaging for the classification of neuropathic facial pain. In this context, neuroimaging refers specifically to structural MRI techniques, including diffusion tensor imaging (DTI) and T1-weighted sequences, which enable the in vivo assessment of gray and white matter alterations associated with pain conditions. Despite these advances, a persistent challenge remains the objective differentiation of neuropathic facial pain subtypes, as clinical diagnosis continues to rely primarily on patient-reported symptomatology. To address this limitation, AI models applied to MRI-derived features have been developed to distinguish between subtypes of neuropathic facial pain and to discriminate affected patients from healthy controls. Due to this retrospective framework, DTI and T1-weighted MRI data were analyzed using RF and LR classifiers in a cohort of 371 adults with TN (265 classical TN (CTN), 106 trigeminal neuropathic pain (TNP), and 108 healthy controls). The results were surprising, with AI models achieving high diagnostic performance, distinguishing CTN from healthy controls with up to 95% accuracy and TNP from healthy controls with up to 91% accuracy. Both classifiers identified gray- and white-matter biomarkers—including cortical thickness, surface area, volume, and diffusivity measures—that significantly differed between groups. As previously shown, classification between CTN and TNP yielded limited accuracy (51%), while revealing differential involvement of the insula and orbitofrontal cortex. Thus, the surprisingly low accuracy in distinguishing CTN from TNP highlights the persistent difficulty of AI-based subtype differentiation, suggesting that current MRI-derived features may be insufficient to capture the full pathophysiological complexity of neuropathic facial pain subtypes.

Further, the progress of DL algorithms—particularly CNNs—in the diagnosis of TN has advanced through a pioneering study that applied DL to identify skull differences in patients with TN, distinguishing them from healthy individuals [103]. The study conducted by Han et al. [104] demonstrated that plain X-ray imaging may serve as an adjunct to conventional MRI—ideally incorporating CISS sequences (Constructive Interference in Steady State)—to support the clinical diagnosis of TN. Using a DenseNet-121 architecture trained on 664 matched subjects, the model achieved an accuracy of 87.2%, with a sensitivity of 0.72, specificity of 0.91, and an AUC of 0.90 on the internal test set.

Another major importance of this technological leap becomes evident when considering that over 60% of patients with TN experience misdiagnoses, often resulting in significant delays in accurate assessment. Many of these patients undergo unnecessary interventions, particularly dental procedures, as frequently reported in the literature up to the early 2020s [105]. In this context, the integration of DL-based diagnostic tools has the potential to substantially improve diagnostic accuracy, reduce inappropriate treatments, and accelerate the initiation of targeted therapeutic strategies.

- Neurovascular pain

The challenge of pain in the orofacial region of neurovascular origin lies in its clinical similarity, in certain cases, to odontogenic pain [106], and this diagnostic dilemma for neurovascular orofacial pain (NVOP) frequently leads a substantial number of patients with migraine or trigeminal autonomic cephalalgias to seek unnecessary dental treatment [107].

Modern, revolutionary diagnostic methods for NVOP disorders in this critical region are progressively moving away from a purely symptom-based diagnostic approach toward more objective and comprehensive frameworks that integrate high-resolution imaging techniques and molecular analyses [108], such as High-Resolution 3D MRI Sequences (3D CISS/FIESTA/SPACE), Magnetic Resonance Angiography (MRA), 3 Tesla Magnets [109], Magnetic Resonance Neurography (MRN) [110], Salivary and Synovial Biomarkers [111], diffusion tensor imaging (DTI) with tractography [112], and last but not least, the remarkable integration of AI technologies both in AI-powered decision-support systems such as GPT-4 [113] and in craniofacial neuroimaging [114].

The category of neurovascular pain of orofacial origin in the current literature includes multiple conditions, classified under the International Classification of Head Disorders (ICHD-3) [115]. Section 5 provides examples of OFPs resembling presentations of primary headache disorders. These conditions occur within the trigeminal nerve distribution (most commonly involving the V2 or V3 branches) [116] and are typically characterized by a vascular-type pain quality, described as throbbing or pulsating.

Among NVOP conditions, migraine represents the most prevalent and clinically relevant entity [117], presenting with a characteristic episodic pattern within the trigeminal distribution and frequently overlapping symptomatically with odontogenic pain, which contributes to diagnostic uncertainty in orofacial settings. Current evidence implicates activation of the trigeminal vascular system as a central mechanism [118], while both genetic susceptibility and environmental influences modulate individual vulnerability to disease expression [119].

Migraine is classified into distinct subtypes, with the most prevalent being migraine without aura and migraine with aura; the latter is characterized by transient neurological symptoms, including visual, sensory, or speech disturbances [120]. Clinicians use the ICHD criteria to categorize the type of migraine [115], but recent advancements in ML offer a robust, data-driven framework for automating diagnosis, identifying specific migraine subtypes. Thus, recent experimental findings demonstrate that the proposed ML framework surpasses conventional diagnostic approaches in terms of classification accuracy, sensitivity, and specificity [121].

The need for non-experimental, patient-based applications of migraine classification, however, was marked by the study of García-Chimeno et al. [122], which represented the first methodological turning point in the field; unlike experimental paradigms or purely imaging-based exploratory work, this study directly analyzed human participants (subjects with chronic migraine with medication overuse, subjects with sporadic/episodic migraine, and healthy controls) using a combined dataset of DTI, from which fractional anisotropy (FA) metrics were extracted as primary biomarkers, together with objective neuroimaging (MRI) and psychological and clinical questionnaires, thereby pioneering an integrated, multimodal diagnostic approach. The authors combined four independent selection algorithms—Gradient Tree Boosting, L1-regularization, RF, and Univariate filtering—into a consensus committee that retained only features repeatedly selected across methods (features repeated more than two or more times). This strategy substantially improved robustness and reduced noise in a small, heterogeneous dataset integrating DTI-derived white-matter metrics and psychological/clinical questionnaire scores. When the committee-refined feature set was used for classification, diagnostic accuracy increased markedly: Naive Bayes improved from 67% to 93%, SVM from 90% to 95%, and boosting from 93% to 94%. The most discriminative features reflected both pain-related behavioral variables (analgesic use, pain days, medication dependence) and structural markers such as left uncinate fasciculus integrity, linking emotional regulation pathways with migraine pathology. So far, this work anticipates that multimodal, ML-driven migraine classification is feasible, clinically applicable, and significantly enhanced by ensemble feature selection strategies, although the small and heterogeneous sample limits the generalizability of these findings to broader migraine populations.

Five years after the pioneering patient-based ML study, Hindiyeh et al. [123] advanced the field by shifting the focus from classification to real-world score characterization within EHRs. The work addressed a different but equally critical challenge: the extraction and validation of soft clinical findings from routine care data, where the documentation is reported to be heterogeneous, incomplete, and largely unstructured. In fact, the authors developed a 10-point migraine model based on clinically meaningful features—headache severity, descriptors, and associated symptoms—defined by two headache specialists and tailored to the realities of EHR documentation. Using NLP and ML algorithms, the authors automated the extraction of 11 key parameters from 2006 encounters and validated these against a manually curated reference standard. The results exceeded initial expectations: automated extraction from unstructured EHR narratives achieved an average F1 score of 92%, far surpassing structured-data extraction (32%). The final migraine outcome score showed 77.2% exact agreement and 82.2% close agreement with human annotators, exceeding the predefined 70% accuracy threshold. Compared with Garcia-Chimeno et al. [122], which relied on imaging and questionnaire data collected under controlled research conditions, the 2022 model represents other major methodological progression: it demonstrates that AI can reliably interpret real-world, clinician-generated text to quantify migraine severity and progression at scale.

In parallel with the advances in EHR-based outcome extraction demonstrated so far, the multicenter study by Cowan et al. [124] provided an important complementary step by validating a computer-based diagnostic engine (CDE) directly against the clinical gold standard of a semi-structured interview conducted by headache specialists. Using ICHD-3 criteria as the reference framework, the authors showed that a self-administered, rule-based online engine could achieve almost perfect diagnostic concordance with expert evaluation and high diagnostic accuracy, with 89% sensitivity and 97% specificity for migraine/probable migraine. This performance exceeded that of earlier digital tools, which were often limited by non-ICHD criteria, retrospective datasets, or lack of comparison with live clinical interviews. Importantly, the CDE reproduced the logic of specialist-driven diagnostic reasoning through mixed chaining and case-based analysis, demonstrating that structured, algorithmic questioning can approximate the cognitive processes underlying expert migraine diagnosis. However, it should be noted that the rule-based architecture of the CDE limits its adaptability to atypical or comorbid presentations frequently encountered in real-world clinical practice.

The next study identified in our research is a 2024 investigation by Khan et al. [125], whose novelty lies primarily in its conceptual shift within the migraine classification literature, as it is the first to introduce a true multiclass framework capable of distinguishing seven distinct migraine subtypes. Their model distinguishes between typical aura with migraine, migraine without aura, typical aura without migraine, familial hemiplegic migraine, sporadic hemiplegic migraine, basilar-type aura, and an additional heterogeneous “other” category. The study demonstrates that, by employing a synthetic data augmentation strategy to expand and balance the clinical dataset, a DL model—specifically a DNN optimized with a dense layer of 512 hidden neurons—achieves a superior classification accuracy of 99.66%, effectively surpassing traditional ML models in the precise differentiation of migraine subtypes. Methodologically, the transition from traditional ML approaches to DL represents a major advancement in modeling capacity and predictive power. Equally relevant, the fourth study from our research moves away from financially demanding neuroimaging techniques, which are often inaccessible, to easily obtainable clinical variables, thereby increasing the feasibility of large-scale deployment, particularly in resource-limited and underdeveloped countries.

### 3.3. Summary of Findings

The working hypothesis of the current research is confirmed, as the reviewed studies consistently show heterogeneous diagnostic performance across odontogenic and non-odontogenic pain categories, reflecting differences in underlying pathophysiology and data complexity. The characteristics of all 20 included studies—comprising study design, sample size, AI algorithm(s) employed, diagnostic criteria, input variables, and diagnostic performance metrics—are summarized in Table 2, organized chronologically within each OFP category.

Across the included studies, a subset of approximately one-third demonstrated high diagnostic performance, defined by accuracy values ≥ 90%, AUC ≥ 0.90, or combined sensitivity and specificity exceeding 85%. These high-performing models were predominantly observed in neurovascular and musculoskeletal pain subdomains, particularly in migraine classification and TMJD pain prediction. Notably, DL architectures, especially DNNs and CNNs, consistently achieved the highest performance metrics, with reported accuracies reaching up to 99.66% in migraine subtype classification [122], and above 95% in musculoskeletal [90] and dental pain [63] applications.

In contrast, classical ML models such as RFs and DT exhibited more variable performance, particularly in terms of precision and sensitivity, highlighting their dependence on feature selection quality and dataset structure. SVMs demonstrated comparatively stable performance across multiple pain subtypes, with consistently high accuracy in both odontogenic and neurovascular domains.

Despite these promising findings regarding the application and practical integration of AI methodologies across OFP subdomains, the present review also highlights that the current body of evidence remains predominantly exploratory, methodologically heterogeneous, and insufficiently standardized to support direct clinical translation. However, the robustness, external validity, and reproducibility of the reported AI models remain limited by several structural and methodological constraints inherent to the available literature.

The critical analysis of the included studies also revealed that AI performance is highly context-dependent, with substantial variability observed across pain categories, imaging modalities, feature selection strategies, and model architectures. Although some studies report high diagnostic accuracy, often exceeding 90% in selected migraine classification or TMD pain prediction models, these results are frequently derived from relatively small datasets, highly selected patient populations, or synthetic data augmentation strategies. Such methodological characteristics may lead to an artificial inflation of performance metrics and consequently limit their generalizability to real-world clinical settings.

A further methodological concern identified during the critical appraisal of the eligible studies relates to class imbalance, which was present either explicitly or implicitly in several datasets. For example, Haddad et al. [63] reported an accuracy of 96.63% on a sample of only 76 patients. Similarly, Garcia-Chimeno et al. [122] achieved SVM accuracy values of 95% on a cohort of merely 52 subjects, while Nam et al. [90] reported a specificity of 99.3% but a markedly lower sensitivity of 69%, suggesting uneven class representation. Notably, Freitas et al. [58], explicitly employed Synthetic Minority Oversampling Technique (SMOTE) to synthetically balance the dataset, indicating that the original class distribution was insufficient for reliable model training. These findings highlight that class imbalance remains an underreported yet influential source of bias in OFP-AI research, warranting cautious interpretation of high-performance values and emphasizing the need for transparent reporting of class distributions in future studies.

From a methodological perspective, another limitation of the included studies is the absence of standardized reporting frameworks for AI performance in OFP research. In many cases, diagnostic accuracy is reported inconsistently, with selective emphasis on favorable metrics (e.g., AUC or accuracy) while underreporting calibration performance, external validation, or failure cases. Furthermore, several studies lack independent external validation cohorts, thereby increasing the risk of overfitting and optimistic performance estimation.

Regarding the limitations of the present review, it should be noted that no formal inter-reviewer agreement metrics (e.g., Cohen’s kappa) were calculated. Nevertheless, study selection and data extraction were performed independently by two reviewers, with disagreements resolved through discussion and adjudication by a third reviewer. This approach likely reduced selection bias, although the level of agreement between reviewers cannot be quantitatively assessed. Beyond reviewer-level limitations, several forms of bias are consistently present across the included literature. First, selection bias is prominent, as many datasets originate from tertiary referral centers, which may not reflect the full clinical spectrum of OFP conditions encountered in general practice. Second, reporting bias is evident, with a tendency to publish studies demonstrating positive or improved AI performance, while negative or inconclusive results are underrepresented in the literature. A relevant example is the study by Stillhart et al. [61], which, although not included in the final selection due to the eligibility criteria, represents one of the few studies reporting limited diagnostic performance of AI systems in pain assessment, as the AFC software failed to detect significant facial expression changes despite variations in self-reported pain levels. Third, spectrum bias is particularly relevant in pain-related AI models, as algorithms are frequently trained on clinically homogeneous or pre-classified populations, limiting their applicability to undiagnosed or mixed-presentation cases.

Moreover, methodological heterogeneity remains one of the most important barriers to evidence synthesis in this field. Variations in pain classification systems, inconsistent use of diagnostic reference standards, and differences in feature extraction approaches (clinical, imaging, neurophysiological, or behavioral data) significantly limit the feasibility of meaningful quantitative synthesis. In principle, a meta-analytic approach would provide a valuable opportunity to generate higher-level evidence and more precise estimates of diagnostic performance across AI models in OFP. However, the current heterogeneity in study design, outcome definitions, and performance reporting limits the reliability and clinical interpretability of such pooled analyses.

Although the ROBIS assessment [38] confirmed a low overall risk of bias across all evaluated domains (Supplementary Material Table S1), the absence of primary study appraisal and pooled robustness testing—inherent to the narrative review design—is transparently reflected in items 3.4 and 4.5 of the assessment and should be considered when interpreting the findings of the present review.

Across all OFP subdomains, an additional structural limitation is the persistent reliance on subjective pain assessment as ground truth labeling for AI training. Given the inherently subjective nature of pain, most datasets depend on patient-reported outcomes (e.g., VAS, NRS, questionnaires), which introduce measurement variability and reduce label reliability. This issue is further compounded by the absence of validated objective biomarkers capable of consistently differentiating pain etiologies across odontogenic, musculoskeletal, neuropathic, and neurovascular conditions.

Despite these shortcomings, current evidence suggests that AI holds substantial promise in supporting diagnostic reasoning, particularly in musculoskeletal pain conditions such as TMDs, as well as in neurovascular nociceptive disorders such as migraine, where multimodal datasets and structured diagnostic frameworks (e.g., ICHD criteria) are comparatively well established. In contrast, odontogenic and neuropathic pain domains remain relatively underdeveloped in terms of AI diagnostic performance, reflecting both limited availability of high-quality datasets and greater clinical heterogeneity.

## 4. Conclusions

This review demonstrates that AI has achieved uneven but increasingly meaningful progress in the diagnostic evaluation of OFP. The highest and most consistent diagnostic performances were observed in neurovascular pain, where well-structured clinical data and standardized diagnostic criteria enabled DL and NLP models to reach accuracy levels approaching near-perfect classification. Musculoskeletal pain assessment using AI algorithms has also shown robust and reproducible results across multiple studies, supported by objective clinical measurements and established diagnostic approaches.

In contrast, diagnostic performance of AI-based models in odontogenic and neuropathic pain was more variable across studies. Notably, although the number of studies investigating odontogenic pain was comparable to that of musculoskeletal pain within the examined decade, the diagnostic performance of AI models in this domain was substantially lower, with high accuracy achieved only in selected imaging-based applications. Other approaches relying on physiological signals, facial expression analysis, or postoperative prediction yielded moderate or limited results. NP models demonstrated moderate to high accuracy in selected radiomics-based studies, but overall outcomes remained inconsistent due to heterogeneous phenotypes and the lack of reliable objective biomarkers.

Overall, the current evidence indicates that AI has the potential to enhance diagnostic accuracy and support clinical decision-making in OFP, particularly within neurovascular and musculoskeletal domains. However, its clinical readiness remains constrained by methodological heterogeneity, small and imbalanced datasets, and limited external validation. Future research should focus on integrating different types of data, using clear and consistent diagnostic criteria aligned with ICOP, and building large, multicenter datasets. These steps pave the way for the development of AI-based diagnostic tools that are reliable, generalizable, and useful in real clinical practice.

## Figures and Tables

**Table 1:** Summary of search strategy, study design, and data sources.

Component	Specification
Databases	PubMed/MEDLINE, Scopus, Web of Science
Timeframe	January 2016–March 2026
Design	Standardized keyword search across databases
Search framework	AI concepts combined with orofacial pain domain terms
AI concept block	ML, DL, CNN, radiomics, computer vision, predictive/diagnostic/classification approaches
Clinical domain block	Spectrum of OFP conditions (odontogenic, and non-odontogenic, TMJD, neuropathic, neurovascular, and related entities)
Coverage strategy	Broad inclusion of etiologies and pain subtypes
Supplementary search	Reference list screening of included studies and systematic reviews

**Table 2:** Included studies organized by OFP category.

Author, Year	Type of Data	Algorithm(s)	Diagnostic Criteria	Dataset Size	Features for Training	Performance
**Odontogenic Pain**						
Teichmann et al., 2020 [60]	Medical records	SVM, ANN, RF	PPG amplitude, wavelet level energies for ECG	47	Self-reported pain during periodontal probing	AUC: 0.811; Se: 71%; Sp: 70%; Acc: 70%
Hu et al., 2019 [62]	fNIRS; AR-based visualization	CNN	Hypersensitive teeth	21	Pain during thermal stimulation	Acc: 73.2%; Se: 54%; Sp: 78.2%
Teichmann et al., 2018 [65]	Medical records	RF	Ramfjord teeth and incisors without crowns	20	Pain during periodontal probing	AUC: 0.828; Se: 87%; Sp: 63%
Haddad et al., 2022 [63]	Medical records	ANN	Vascular dynamics in inflammatory tooth using infrared thermography	76	Painful symptoms vs. asymptomatic	Acc: 96.63%; Se: 98.69%; Pre: 95.87%
Nosrat et al., 2023 [59]	Clinical data	RF	NSRetx	3666	Moderate-severe pain ± swelling 14 days post-NSRetx	Acc: 82%; Se: 49%; Sp: 83%; Pre: 13%
Freitas et al., 2026 [58]	Clinical data	LR, SVM, GB, RF, DT, KNN, AdaBoost, MLP	Pain after endodontic treatment at 24 h and 72 h	354	Positive cold test response; persistent discomfort after stimulus removal	24 h: AUC 74%, Pre 81%; 72 h: AUC 81%, Pre 88%
**Non-Odontogenic Pain**						
***Musculoskeletal Pain***						
McCartney et al., 2013 [88]	Online questionnaire data	ANN	Classification scheme for facial pain syndromes; binomial questionnaire	607	Responses to updated online facial pain questionnaire	TN1: Se 92.4%, Sp 62.5%; TN2: Se 87.8%, Sp 96.4%; TNP: Se 86.7%, Sp 95.2%; TDP: Se 0%, Sp 100%; Symptomatic TN: Se 100%; PHN: Se 100%; NIN: Se 50%, Sp 99%; AFP: Se 0%, Sp 99%; GPN: Se 0%, Sp 100%; TMJ: Se 0%, Sp 99%
Nam et al., 2018 [90]	Medical records	NLP	RDC/TMD	260	Maximum mouth opening	Acc: 96.6%; Se: 69%; Sp: 99.3%
Nocera et al., 2021 [91]	Medical records	HFV Algorithm	TMJD Pain Screener questionnaire	451	Large datasets for ML models and data augmentation	Acc: 95.55%; Se: 95%
Yildiz et al., 2024 [93]	Medical records	MARS	Shapiro–Wilk test; Levene’s test	228	Maximum mouth opening; TMJ lateral excursion	Acc: 89.66%; AUC: 93%; F1: 90.32%
Zatt et al., 2024 [94]	Clinical records	DT	Joint or muscle disorders	122	Clicking; mouth opening; temporal muscle palpation pain	Joint disorders: Acc 84%, F1 85%; Myofascial disorders: Acc 78%, F1 76%
De Araujo et al., 2024 [95]	Self-reported symptoms	NLP	TMJD Pain Screener questionnaire	50	Identification and differentiation of pain source	Acc: 86%
Lee et al., 2025 [96]	Self-reported symptoms	myTMJ mobile app	TMJD Pain Screener questionnaire	110	Jaw pain; headache; clicking jaw; facial pain	Acc: 95.5%
***Neuropathic Pain***						
Mulford et al., 2022 [102]	Medical records	CNN	ICHD-3 criteria	134	Texture and morphological features from MRI related to TN	AUC: 0.83; Acc: 78%; Se: 82%; Sp: 76%
Latypov et al., 2023 [103]	Medical records	RF, LR	MRI images	479	Gray and white matter-based predictive metrics related to TN	RF: Acc 86%; LR: Acc 95%
Han et al., 2025 [104]	Clinical data	CNN	X-ray skull images	664 (166 TN; 498 controls)	TN vs. unruptured intracranial aneurysms	Acc: 87.2%; Se: 72%; Sp: 91%
***Neurovascular Pain***						
Garcia-Chimeno et al., 2017 [122]	Medical records	SVM, AdaBoost, NB	MRI changes in white and gray matter; questionnaires for migraine detection	52	Large datasets for ML models and data augmentation	SVM: Acc 95%, Pre 93%, F1 92%; AdaBoost: Acc 94%, Pre 96%, F1 92%; NB: Acc 93%, Pre 90%, F1 92%
Hindiyeh et al., 2022 [123]	Medical records	NLP, SQL	Migraine model scores	2006	Symptoms	Acc: 82.2%; Sp: 72%; F1: 92%
Cowan et al., 2022 [124]	Medical records	CDE	ICHD-3 criteria	266	Web-based expert questionnaire and analytics	Acc: 91.6%; Se: 89%; Sp: 97%
Khan et al., 2024 [125]	Medical records	DNN, SVM, KNN, RF, DT	Python-based Migraine classification framework	400	Large datasets for ML models and data augmentation	DNN: 99.66%; SVM: 94.60%; KNN: 97.10%; DT: 88.20%; RF: 98.50%

## Data Availability

No new data were created or analyzed in this study. Data sharing is not applicable to this article.

## Supplementary Materials

The following supporting information can be downloaded at: https://www.mdpi.com/article/10.3390/diagnostics16121801/s1, Table S1: ROBIS Assessment.

## Abbreviations

The following abbreviations are used in this manuscript:

Acc	Accuracy
AFP	Atypical facial pain
AI	Artificial intelligence
AR	Augmented reality
AdaBoost	Adaptive boosting
ANNs	Artificial neural networks
AUC	Area under the curve
AFC	Automated face coding
CBCT	Cone–beam computed tomography
CNNs	Convolutional neural networks
CDE	Computer-based diagnostic engine
CISS	Constructive Interference in Steady State
CTN	Conventional trigeminal neuralgia
DL	Deep learning
DTI	Diffusion tensor imaging
DC/TMD	Diagnostic Criteria for Temporomandibular Disorders
DT	Decision tree
DNN	Deep neural network
EDA	Electrodermal activity
EMG	Electromyography
EEG	Electroencephalography
EHRs	Electronic health records
ECG	Electrocardiography
FIESTA	Fast imaging employing steady-state acquisition
fMRI	Functional magnetic resonance imaging
fNIRS	Functional near-infrared spectroscopy
GB	Gradient boosting
GPN	Glossopharyngeal neuralgia
F1	F1 score
HFV	High frequency value
HRV	Heart rate variability
ICOP	International Classification of Orofacial Pain
ICHD-3	International Classification of Head Disorders
KNN	K-nearest neighbors
LDF	Laser Doppler flowmetry
LSTM	Long short-term memory
LR	Logistic regression
ML	Machine learning
MLP	Multilayer perceptron
MMD	Masticatory muscle disorders
MARS	Multivariate adaptive regression splines
MRN	Magnetic resonance neurography
MRI	Magnetic resonance images
MRA	Magnetic resonance angiography
NB	Naive Bayes
NIN	Nervus Intermedius Neuralgia
NRS	Numerical rating scale
NLP	Natural language processing
NN	Neural network
NSRetx	Nonsurgical root canal retreatment
NVOP	Neurovascular orofacial pain
PHN	Postherpetic neuralgia
PO	Pulse oximetry
Pre	Precision
PR	Precision recall
PPG	Photo plethysmogram
OFP	Orofacial pain
RF	Random Forest
ROC	Receiver operating characteristic
RDC/TMD	Research Diagnostic Criteria for Temporomandibular Disorders
RNNs	Recurrent neural networks
Se	Sensitivity
Sp	Specificity
SQL	Structured query language
SVM	Support vector machine
SCL-90-R	Symptom checklist-90-revised
SMOTE	Synthetic minority oversampling technique
TMJ	Temporomandibular joint
TNP	Trigeminal neuropathic pain
TMJD	Temporomandibular joint disorders
TN	Trigeminal neuralgia
TDP	Trigeminal deafferentation pain
VAS	Visual analog scale
VRS	Verbal rating scale
QST	Quantitative sensory testing
