# Supplementary material for: Artificial Intelligence in Orofacial Pain: Diagnostic and Predictive Performance Across Machine Learning and Deep Learning Models
Source: Diagnostics (Basel). 2026 Jun 11;16(12):1801. doi: 10.3390/diagnostics16121801 (PMC13298713; doi:10.3390/diagnostics16121801)
Supplement: Supplementary file 1 [file diagnostics-16-01801-s001.zip › diagnostics-4356354-supplementary.pdf]

Supplementary Material — ROBIS Assessment

**PHASE 1: Assessing Relevance (DTA Review)**			
—	Does the question addressed by the review match the target question (diagnostic performance of AI models in OFP)?	**Y**	The review explicitly addresses diagnostic and predictive performance of ML and DL models across the main OFP categories, consistent with DTA review framework.
**DOMAIN 1: Study Eligibility Criteria**			
1.1	Did the review adhere to pre-defined objectives and eligibility criteria?	**Y**	Objectives and eligibility criteria were pre-specified and clearly stated in Section 2.3 prior to study selection.
1.2	Were the eligibility criteria appropriate for the review question?	**Y**	Inclusion criteria restricted to human studies employing at least one AI method for OFP diagnosis/prognosis, with at least two diagnostic performance metrics.
1.3	Were eligibility criteria unambiguous?	**Y**	Inclusion and exclusion criteria listed as explicit, non-overlapping bullet points in Section 2.3.
1.4	Were restrictions based on study characteristics appropriate?	**Y**	Restricted to full-text, English-language original studies published 2016–2026. Pediatric and non-stomatognathic pain studies excluded.
1.5	Were restrictions based on sources of information appropriate?	**Y**	Three major international databases selected (PubMed/MEDLINE, Scopus, Web of Science), supplemented by manual reference screening.
	**Concerns regarding specification of study eligibility criteria**	**LOW**	Eligibility criteria were pre-specified, unambiguous, and appropriate to the review question.
**DOMAIN 2: Identification and Selection of Studies**			
2.1	Did the search include an appropriate range of databases/electronic sources?	**Y**	Search conducted in PubMed/MEDLINE, Scopus, and Web of Science — three major international databases with complementary indexing coverage.
2.2	Were methods additional to database searching used?	**Y**	Manual screening of reference lists of all included studies and relevant systematic reviews was performed to identify additional eligible records.
2.3	Were search terms likely to retrieve as many eligible studies as possible?	**Y**	A comprehensive, structured Boolean search string combining AI concepts (ML, DL, CNN, radiomics, predictive

			modelling) with the full spectrum of OFP conditions was applied uniformly across all databases (Section 2.2, Table 1).
2.4	Were restrictions based on date, publication format, or language appropriate?	**Y**	Timeframe (2016–2026) selected to capture the most recent AI evidence. English-language restriction appropriate given indexed literature in the field.
2.5	Were efforts made to minimise error in selection of studies?	**Y**	Title/abstract and full-text screening performed independently by two reviewers (A.G.W. and A.A.M.C.); disagreements resolved by a third reviewer (L.I.).
	**Concerns regarding methods used to identify and/or select studies**	**LOW**	Comprehensive, multi-database search with supplementary reference screening and dual independent reviewer selection process.
**DOMAIN 3: Data Collection and Study Appraisal**			
3.1	Were efforts made to minimise error in data collection?	**Y**	Data extraction performed using a standardized form by two independent reviewers. Extracted variables included study design, population, pain type, AI algorithm, and diagnostic performance metrics.
3.2	Were sufficient study characteristics available for interpretation of results?	**Y**	All included studies were summarized in a comprehensive table covering author data, study design, type of data used, pain category, AI method, dataset characteristics, and key performance metrics.
3.3	Were all relevant study results collected for use in the synthesis?	**Y**	All reported diagnostic performance metrics (accuracy, sensitivity, specificity, AUC, F1-score, precision, recall) were extracted and included.
3.4	Was risk of bias formally assessed using appropriate criteria?	**PN**	Formal risk of bias assessment of primary studies was not performed, consistent with the narrative review design. Methodological limitations of included studies are discussed qualitatively in the relevant sections.
3.5	Were efforts made to minimise error in risk of bias assessment?	**NI**	Not applicable; formal risk of bias assessment of primary studies was not conducted given the narrative review design.
	**Concerns regarding methods used to collect data and appraise studies**	**LOW**	Data collection was rigorous and standardized. The absence of formal primary study RoB assessment is

			acknowledged and consistent with the narrative review design.
**DOMAIN 4: Synthesis and Findings**			
4.1	Did the synthesis include all studies that it should?	**Y**	All 21 studies identified through the selection process were included in the narrative synthesis, organized by OFP category.
4.2	Were all pre-defined analyses reported or departures explained?	**Y**	Findings were organized according to the four pre-defined OFP categories (odontogenic, musculoskeletal, neurovascular, neuropathic), consistent with the stated review framework.
4.3	Was the synthesis appropriate given study designs and outcomes?	**Y**	Narrative synthesis was appropriate given the methodological heterogeneity of included studies (different AI methods, datasets, pain types, and performance metrics), which precluded quantitative pooling.
4.4	Was between-study variation (heterogeneity) minimal or addressed?	**PY**	Heterogeneity was addressed narratively and acknowledged as a limitation, including variability in study populations, AI algorithms, and performance reporting across included studies.
4.5	Were findings robust (e.g. funnel plot or sensitivity analyses)?	**NI**	Not applicable; quantitative robustness analyses (funnel plot, sensitivity analysis) are not applicable in the context of a narrative review.
4.6	Were biases in primary studies minimal or addressed in the synthesis?	**PY**	Potential biases in primary studies (small sample sizes, single-centre designs, lack of external validation, class imbalance) were identified and discussed qualitatively.
	**Concerns regarding the synthesis and findings**	**LOW**	Narrative synthesis was appropriate and well-justified. Heterogeneity and primary study limitations were acknowledged.
**PHASE 3: Overall Risk of Bias**			
A	Did the interpretation of findings address all concerns identified in Domains 1–4?	**Y**	Limitations identified across all domains are discussed in the Limitations section.
B	Was the relevance of identified studies to the review's research question appropriately considered?	**Y**	All included studies were directly relevant to the diagnostic/predictive performance of AI in OFP. Non-relevant studies were excluded per pre-defined criteria.

C	Did the reviewers avoid emphasizing results on the basis of their statistical significance?	**Y**	Results were reported comprehensively across all performance metrics; studies with moderate performance were included and discussed alongside high-performing models.
	**OVERALL RISK OF BIAS IN THE REVIEW**	**LOW**	The review demonstrates a transparent and rigorous process across all assessed domains. The narrative design is clearly stated and consistently applied throughout.

Y = Yes | PY = Probably Yes | PN = Probably No | N = No | NI = No Information
